# Application of pulsed-field gel electrophoresis to an international outbreak of Salmonella agona.

**DOI:** 10.3201/eid0202.960209

**Published:** 1996

**Authors:** E J Threlfall, M D Hampton, L R Ward, B Rowe

**Affiliations:** Laboratory of Enteric Pathogens, Central Public Health Laboratory, London, United Kingdom.


### Application of Pulsed-Field Gel Electrophoresis to an International Outbreak of *Salmonella agona*

Between 1 December 1994 and 31 January 1995, *Salmonella agona* infections increased abruptly in England and Wales; isolates of *S. agona* from 41 patients with diarrheal illness were referred to the Laboratory of Enteric Pathogens of the Public Health Laboratory Service, compared with nine cases in the previous 12 months. Most isolates were from children under 10 years of age. Many of the cases were in Jewish children in London and several other parts of England; two children required hospital admission. *S. agona* can be subdivided by phage typing and, by using a method developed in the Laboratory of Enteric Pathogens (L. R. Ward, unpublished manuscript), we found that the isolates belonged to *S. agona* phage type (PT) 15. The outbreak was traced to a kosher savory snack imported into the United Kingdom from Israel, and all isolates from the contaminated product and from patients who had a history of consuming this product were found to belong to *S. agona* PT 15 (1). (Full details of this outbreak will be published elsewhere.)

After notification of the outbreak in the United Kingdom, health authorities in countries where the contaminated snack had also been distributed were warned of the possibility of an upsurge in *S. agona* infections; these countries included Israel, the United States, and Canada. Countries in the European Union (EU) were also notified through SALM-NET, the European salmonella surveillance network (2). As a result of such notification, isolates of *S. agona* subsequently identified as PT 15 were received from patients in Israel, the United States, and France and from food samples in Israel, the United States, and Canada.

For outbreak investigations, the policy of the Laboratory of Enteric Pathogens is to use serotyping, phage typing, and antibiogram analysis (R-typing) for the primary identification and subdivision of isolates, supplemented when appropriate by a range of DNA-based methods, including plasmid analysis, ribotyping, insertion sequence (IS) *200* fingerprinting, and pulsed-field gel electrophoresis (PFGE). The use of these techniques has recently been reviewed (3). Of these methods, PFGE has been used for the molecular fingerprinting of *S. typhi* (4, 5) and for subdivision within epidemic phage types of *S. enteritidis* (6, 7).

Because a small number of patients in England and Wales had been infected with *S. agona* PT 15 in the 11 months before the kosher snack outbreak, we decided to characterize the outbreak isolates by genotypic methods and, if possible, to use such methods for subdivision within the phage type. For *S. agona*, strains of this serotype do not possess IS*200* elements (8), and studies by the Laboratory of Enteric Pathogens have demonstrated that the serotype is unlikely to be subdivided by ribotyping (M.D. Hampton, E.J. Threlfall, unpublished observations). PFGE was, therefore, considered the method most likely to provide a genotypic fingerprint suitable for epidemiologic investigations. Isolates from the food product and from all patients infected both during the outbreak and in the preceding 11 months were, therefore, examined by PFGE. Similarly, because of the international distribution of the contaminated food product, *S. agona* PT 15 organisms isolated in Israel and Canada from the food product and also from specimens from patients in Israel, the United States, and France were examined by PFGE.

Analysis by PFGE of the fragments resulting from *Xba* I digestion of genomic DNA from 78 isolates of *S. agona* PT 15 made in the United Kingdom, Israel, the United States, Canada, and France between December 1993 and April 1995 showed 11 distinct pulsed-field profiles (PFP) and one variant profile, with 14 to 17 resolvable chromosomal fragments, ranging from approximately 25 kb to 680 kb (Figure). These profile types have been designated *S. agona* PFP (*Xba*I) 1 through to *S. agona* PFP (*Xba*I) 9, and *S. agona* PFPs (*Xba*I) 11 and 12; the variant type has been designated *S. agona* PFP (*Xba*I) 6a. The pattern designated *S. agona* PFP (*Xba*I) 10 had been assigned to an isolate of *S. agona* of an unrelated phage type, which has been used as a control in PFGE analysis of this serotype.

The predominant PFGE profile, *S. agona* PFP (*Xba*I) 6, gave at least 15 resolvable fragments, ranging from 25 kbp to 485 kbp; this profile type was exhibited by 51 of the 78 isolates examined. The PFP 6 variant, designated PFP 6a and identified in one of the Canadian food isolates, could be differentiated from PFP 6 by the presence of an additional fragment of approximately 395 kbp; in

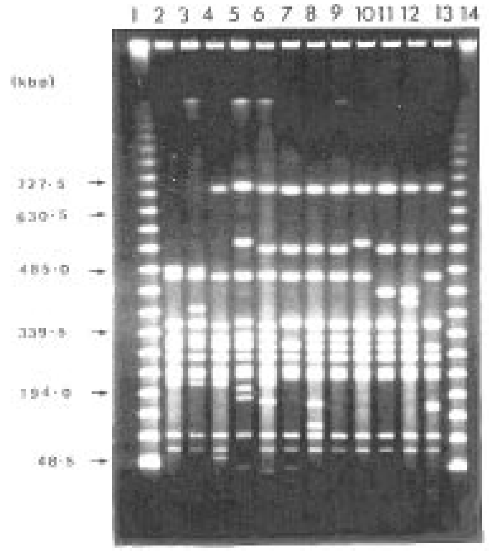

**Figure.** PFGE profiles of *Xba*I-digested genomic DNA from strains of *S. agona* PT 15. *Legend*: Tracks 1 - 14 contained: 1 and 14, lambda 48.5-kb ladder (Sigma); 2, *S. agona* PFP (*Xba*I) 6; 3, PFP 6a; 4, PFP 4; 5, PFP 10 (= control PFP type for *S. agona*); 6, PFP 9; 7, PFP 7; 8, PFP 3; 9, PFP 2; 10, PFP 5; 11, PFP 1; 11, PFP 8; 13, PFP 9. Gels were run at 6.0 V $cm^{-1}$ for 36 h with a 25- to 75-s pulse ramp time.

all other respects, *S. agona* PFP 6a was indistinguishable from *S. agona* PFP 6.

The distribution of *Xba*I-generated PFGE types between source group and country of isolation in isolates of *S. agona* PT 15 is shown in the Table. Of the 10 PFGE profiles in 46 isolates from persons in the United Kingdom, 26 isolates (56.5%) belonged to PFP 6. All isolates with this profile pattern were made between 25 November 1994 and 17 February 1995 and were from patients associated with the kosher snack–related outbreak. Two isolates of *S. agona* PT 15 from the contaminated food product were also examined and both belonged to PFP 6. In contrast, PFP 6 was not identified in any isolates of *S. agona* PT 15 made in the United Kingdom in the 11 months before the outbreak. In one case, *S. agona* PT 15 with the PFGE profile PFP 9 was isolated from a patient just after the outbreak began, in November 1994. However, the PFGE profile of this isolate was sufficiently unrelated to that of the outbreak isolates (PFP 6) to warrant exclusion of the patient from the kosher snack–related cases. Nine PFGE profiles were identified in the remaining 19 U.K. isolates, with PFPs 5, 2, and 1 predominating (Table). Four of five patients infected with isolates with the PFP 2 pattern lived in northwestern England, and three of five patients with the PFP 5 profile lived in southwestern England or southern Wales. None of these nine PFGE profiles were identified in isolates from case-patients associated with the kosher snack outbreak.

Nine isolates of *S. agona* PT 15 from patients and two isolates from the contaminated food product that had been made in Israel were also examined by PFGE and, without exception, all strains belonged to PFP 6. Likewise, all of 10 isolates from outbreak-associated cases in the United States belonged to PFP 6 as did two of three strains isolated in Canada from the contaminated food product; the PFP of the remaining Canadian strain, PFP 6a, was closely related to PFP 6. Three PFPs were identified in the six isolates of *S. agona* PT 15 from France, of which two (PFP 2 and PFP 5) had been observed in isolates of PT 15 made in the United Kingdom before the outbreak. However, none of the isolates of *S. agona* PT 15 made in France were of PFP 6 (Table).

In conclusion, 11 distinct PFGE profile types and one variant type have been identified in 78 isolates of *S. agona* PT 15 made in the United Kingdom, Israel, the United States, Canada, and France between December 1993 and April 1995. One PFGE profile type, PFP 6, was specifically associated with isolates from a contaminated savory snack and from persons who consumed this product in the United Kingdom, Israel, and the United States. These results demonstrate that one strain of *S. agona* PT 15, with a characteristic PFGE profile, was responsible for outbreaks in the United Kingdom, Israel, and the United States in 1994-95. In contrast, the PFGE profile types of isolates of *S. agona* PT 15 identified in the United Kingdom either in the 11 months before the outbreak (or in one case at the start of the outbreak) and of isolates of PT 15 obtained from patients in France were unrelated to PFP 6. We conclude that PFGE fingerprinting provides a genotypic method for subdivision within phage type 15 of *S. agona* and that this method has provided an invaluable supplement to phage typing for investigation of international outbreaks. It is, however, important to realize that PFGE typing may not be applicable to all salmonella serotypes and phage types; thus, the method's results should be carefully evaluated by using both epidemiologically-related and unrelated isolates before it is used for outbreak investigations.

Table. Distribution of *XbaI* pulsed-field profiles in isolates of *Salmonella agona* phage type 15 made in the United Kingdom, Israel, USA, Canada, and France, 1993–1995[a]

| | | Number | Pulsed-field profiles (PFP) | | | | | | | | | | | | |
|---|---|---|---|---|---|---|---|---|---|---|---|---|---|---|---|
| Country | Source | studied | 1 | 2 | 3 | 4 | 5 | 6 | 6[a] | 7 | 8 | 9 | 10 | 11 | 12 |
| United Kingdom | Human | 46 | 3 | 5 | 1 | 2 | 5 | 26[b] | | 1 | 1 | 1 | | 1 | |
| | Snack | 2 | | | | | | 2 | | | | | | | |
| Israel | Human | 9 | | | | | | 9 | | | | | | | |
| | Snack | 2 | | | | | | 2 | | | | | | | |
| United States | Human | 10 | | | | | | 10 | | | | | | | |
| Canada | Snack | 3 | | | | | | 2 | 1 | | | | | | |
| France | Human | 6 | | 1 | | | 4 | | | | | | | | 1 |
| Total | | 78 | 3 | 6 | 1 | 2 | 9 | 51 | 1 | 1 | 1 | 1 | 0 | 1 | 1 |

[a] Isolates from Israel, the United States, and Canada were made in 1995; of the isolates from France, 3 were obtained in 1994 and 3 in 1995.

[b] Isolated 25 November 1994 to 17 February 1995.

**E. John Threlfall, Michael D. Hampton, Linda R. Ward, and Bernard Rowe**
Laboratory of Enteric Pathogens, Central Public Health Laboratory, London, United Kingdom

### Acknowledgments

We are grateful to the following microbiologists for sending strains of *S. agona* for typing: Dr. N. Andorn, Ministry of Health, Government Central Laboratories, Jerusalem, Israel; Dr. Mehdi Shayedgani, New York State Department of Health, New York, NY, USA; Dr. Rasik Khakhria, National Laboratory of Enteric Pathogens, Laboratory Centres for Disease Control, Ottawa, Canada; and Professor Patrick Grimont, Unites des Enterobacteries, Institut Pasteur, Paris, France.

### References

1. An outbreak of *Salmonella agona* due to contaminated snacks. CDR Weekly 1995; 5: 29-32.
2. Fisher IST, Rowe B, Bartlett CLR, Gill ON. “Salm-Net”—laboratory-based surveillance of human salmonella infections in Europe. PHLS Microbiol Digest 1994; 11: 181-2.
3. Threlfall EJ, Powell NG, Rowe B. Differentiation of salmonellas by molecular methods. PHLS Microbiol Digest 1994; 11: 199-202.
4. Thong KL, Cheong YM, Puthucheary S, Koh CL, Pang T. Epidemiologic analysis of sporadic *Salmonella typhi* isolates and those from outbreaks by pulsed-field electrophoresis. J Clin Microbiol 1994; 32: 1135-41.
5. Nair S, Poh CL, Lim YS, Tay L, Yoh KT. Genome fingerprinting of *Salmonella typhi* by pulsed-field gel electrophoresis for subtyping common phage types. Epidemiol Infect 1994; 113: 391-402.
6. Powell NG, Threlfall EJ, Chart H, Rowe B. Subdivision of *Salmonella enteritidis* PT 4 by pulsed-field gel electrophoresis: potential for epidemiological surveillance. FEMS Microbiol Lett 1994; 119: 193-98.
7. Powell NG, Threlfall EJ, Chart H, Schofield SL, Rowe, B. Correlation of change in phage type with pulsed-field profile and 16S rrn profile in *Salmonella enteritidis* phage types 4, 7 and 9a. Epidemiol Infect 1995; 114: 403-11.
8. Gibert I, Barbé J, Casadesus J. Distribution of insertion sequence IS*200* in *Salmonella* and *Shigella*. J Gen Microbiol 1995; 136: 2555-60.
